# Nanoparticles for treatment of bovine *Staphylococcus aureus* mastitis

**DOI:** 10.1080/10717544.2020.1724209

**Published:** 2020-02-08

**Authors:** Samah Attia Algharib, Ali Dawood, Shuyu Xie

**Affiliations:** a National Reference Laboratory of Veterinary Drug Residues (HZAU) and MAO Key Laboratory for Detection of Veterinary Drug Residues, Huazhong Agricultural University, Wuhan, China;; b Department of Clinical Pathology, Faculty of Veterinary Medicine, Benha University, Benha, Egypt;; c The State Key Laboratory of Agricultural Microbiology, (HZAU), Wuhan, China;; d Department of Medicine and Infectious Diseases, Faculty of Veterinary Medicine, University of Sadat City, Sadat City, Egypt;; e MOA Laboratory for Risk Assessment of Quality and Safety of Livestock and Poultry Products, Huazhong Agricultural University, Wuhan, China

**Keywords:** *S. aureus*, mastitis, resistance, drug delivery, nanoparticles, nanogel

## Abstract

*Staphylococcus aureus (S. aureus)* is one of the most important zoonotic bacterial pathogens, infecting human beings and a wide range of animals, in particular, dairy cattle. Globally. *S. aureus* causing bovine mastitis is one of the biggest problems and an economic burden facing the dairy industry with a strong negative impact on animal welfare, productivity, and food safety. Furthermore, its smart pathogenesis, including facultative intracellular parasitism, increasingly serious antimicrobial resistance, and biofilm formation, make it challenging to be treated by conventional therapy. Therefore, the development of nanoparticles, especially liposomes, polymeric nanoparticles, solid lipid nanoparticles, nanogels, and inorganic nanoparticles, are gaining traction and excellent tools for overcoming the therapeutic difficulty accompanied by *S. aureus* mastitis. Therefore, in this review, the current progress and challenges of nanoparticles in enhancing the *S. aureus* mastitis therapy are focused stepwise. Firstly, the *S. aureus* treatment difficulties by the antimicrobial drugs are analyzed. Secondly, the advantages of nanoparticles in the treatment of *S. aureus* mastitis, including improving the penetration and accumulation of their payload drugs intracellular, decreasing the antimicrobial resistance, and preventing the biofilm formation, are also summarized. Thirdly, the progression of different types from the nanoparticles for controlling the *S. aureus* mastitis are provided. Finally, the difficulties that need to be solved, and future prospects of nanoparticles for *S. aureus* mastitis treatment are highlighted. This review will provide the readers with enough information about the challenges of the nanosystem to help them to design and fabricate more efficient nanoformulations against *S. aureus* infections.

## 1. Introduction

Bovine mastitis, generally caused by several different bacteria, is one of the most devastating diseases in dairy herds worldwide (Tiwari et al., 2013; Ruegg, 2017). Among these bacteria, *S. aureus* is a predominant pathogen causing the highest virulent forms of bovine mastitis and strikes the greatest challenge to dairy production in most countries (Monistero et al., 2018). This bacterium causes significant economic losses, including a severe decline in milk revenue, reproductive complications, and expenses incurred from the culling of infected animals, increased costs of veterinary medication, and replacing tainted milk (Hogeveen, 2005; Hogeveen et al., 2011; Deb et al., 2013; Botaro et al., 2015; Gomes & Henriques, 2016). Furthermore, numerous types of toxins and enzymes in the milk produced by *S. aureus* can lead to severe food-borne diseases (Johler et al., 2013). In addition, their persistence in the cells can establish a reservoir for relapsing infection and it is associated with the clinical, subclinical and recurrent infection of bovine mastitis (Zhou et al., 2018).

Antibiotic treatment is considered one of the main measures for mastitis control. The therapeutic effects depend on disease severity, drug choice, reasonable drug usage and dosages, and prohibition of predisposing causes. Treatment of mastitis by antibiotics is still under debate to develop a standard treatment regime to obtain satisfactory effects (du Preez, 2000) due to persistent intracellular existence with different forms protected it from antibiotics and host defense mechanism after that; they can relapse to more infectious wild-type phenotype, probably causing recurrent infection. Besides, large usage of antibiotics for the long-term increasingly leads to the resistance of *S. aureus* to antibiotics (Szweda et al., 2014).

Throughout the previous years, much anxiety has been raised regarding the treatment failure. Consequently, continual attention has given by the researchers to discover new strategies for treatment (Dehkordi et al., 2011; Jamaran & Zarif, 2016). Recently, nano drugs have been used as a substitute measure to solve the multi-drug resistance and intracellular persistence of *S. aureus* which associated with the subclinical and relapsing infection of bovine mastitis (Le Ray et al., 2005; Franci et al., 2015; Wang et al., 2017; Zhou et al., 2018). So, these new nanocarriers provide a new strategy to combat *S. aureus* mastitis problems. In order to provide an overview of the emerging nanocarriers in the bovine mastitis management and help the researcher to understand how they can discover a new trend to combat *S. aureus* mastitis by shifting their attention toward the world of nanocarriers. We searched PubMed, Scopus, and Web of Science for all the studies published over the last 20 years using the keywords “*S. aureus* mastitis” or “virulence factors of *S. aureus*” and “antimicrobial resistance” or “nanoparticles and nanogel”. About 3000 records and 550 of closely related papers were screened for suitable studies. We summarized the features and treatment difficulty of *S. aureus* mastitis, the advantages, and prospects of nanoparticles and nanogels according to the related publications.

## 2. Therapy difficulty of *S. aureus*

The effects of antimicrobial drugs in mastitis treatment depend on its pharmacokinetics, such as its penetration into the milk when infused parenterally, the rate of absorption and distribution of the drug when administered intramamarily, and others. These characters are related to lipid solubility, a degree of ionization, a degree of adherence with serum and mammary gland proteins, and kind of the vehicle (Prescott et al., 2000). The weak organic bases are accumulated in the milk as ionized form after administered parenterally with higher concentrations than present in the blood. Conversely, weak acids concentrations in milk are extensively lower than in blood. The pharmacodynamics are also an important aspect and must be taken into considerations. Whereas, the antibiotics have several modes of action, including preventing bacterial cell wall synthesis, preventing protein synthesis by interfering with ribosome function, inhibiting DNA synthesis (Normark & Normark, 2002), and others.

The cow with infected udder is complicated or even impossible to therapy positively due to: several types from the bacteria have the capability to produce various kinds from enzymes and toxins which lead to udder tissue damage and increase the ability of the microbes to the tissue; surviving of the microorganism in the keratin layer of the teat canal which acts as inhibitory in normal status; some strains have the protein A, this protein binds with Fc portion of the antibody; therefore, the bacteria persist unrecognizable to the neutrophil and it cannot phagocyte them; surviving and multiplication of the bacteria in the phagocytes; approximately 50% of *S. aureus* strains isolated from diseased cattle produce beta-lactamase; as well as, formation of micro-abscesses and atrophy of glandular tissue around the infected site. All these facts make the penetration of the antibiotics to the fibrous membranes is very complicated.

Therefore, the resistance of *Staphylococci* to antibiotic become one of the most massive problems in therapy, predominantly *S. aureus* to penicillin G (Olsen et al., 2006). Coagulase-negative *Staphylococci* tend to be further resistant than *S. aureus* and can progress multi-resistance (Pitkälä et al., 2004). Some researchers discussed that results from susceptibility tests did not associate with cure rates of mastitis (Haveri et al., 2005). The β-lactamase test is used for detecting the resistance of *Staphylococci* to penicillin G to avoid the problem (Olsen et al., 2006). Moreover, a different attitude was later suggested to progress the susceptibility tests of mastitis pathogens (Klement et al., 2005). The bactericidal drug should preferably be used (Kehrli & Harp, 2001) with a low minimum inhibitory concentration (MIC) rate for the target pathogens (Prescott et al., 2000) and not affects milk compositions such as macrolides, tetracyclines, and trimethoprim-sulphonamides.

**Figure 1.**
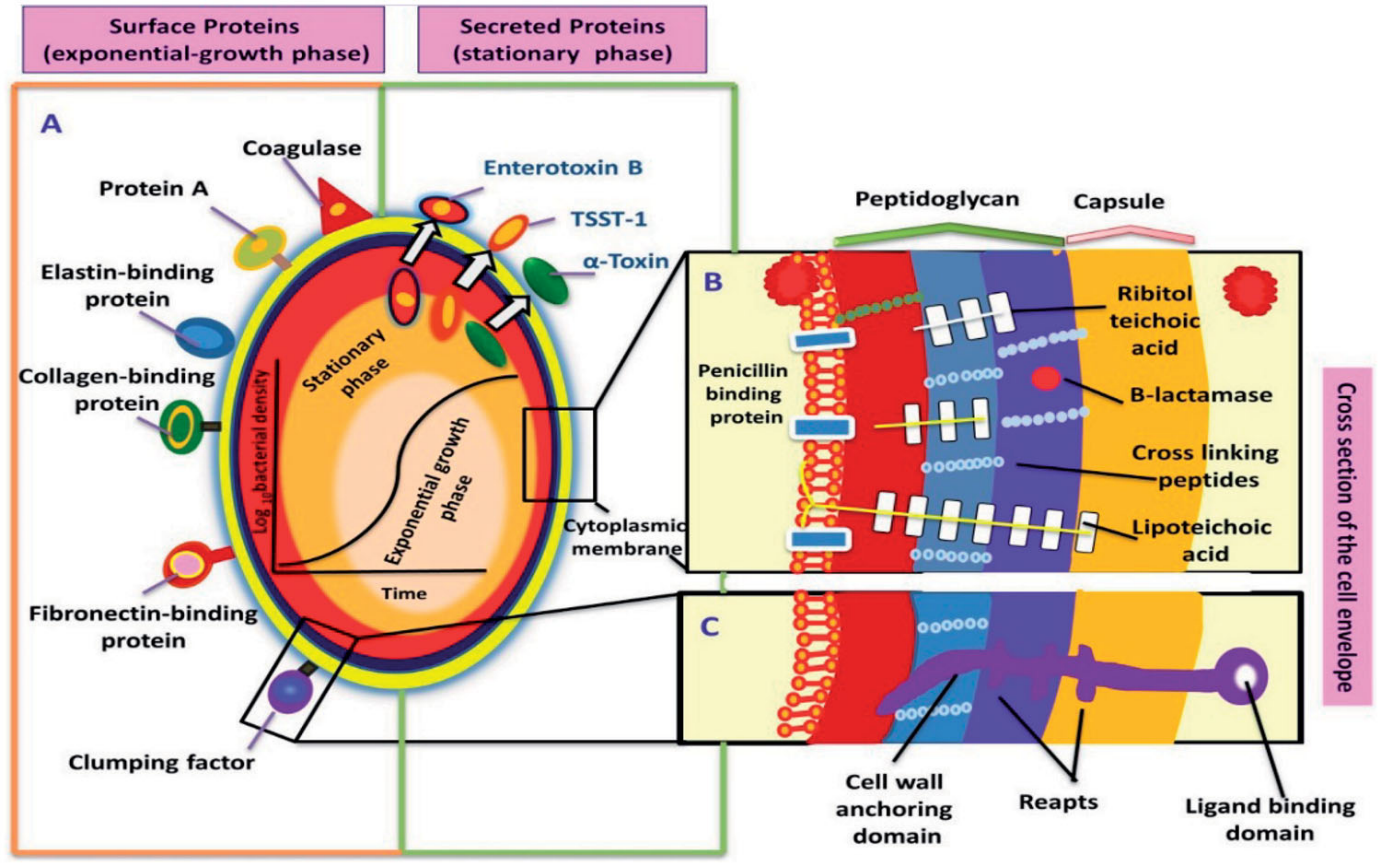
The secreted virulence factors of *Staphylococcus aureus*. (A) the surface and secreted protein, most of these proteins can be created during the growth phase. (B) and (C) show cross-section in the cell envelope. TSST: toxic shock syndrome toxin.

The virulence factors of *S. aureus* are wide-ranging, with both structural and secreted products, playing a fundamental role in the pathogenesis of its infection (Figure 1). Partly selected factors (Matsunaga et al., 1993; Dinges et al., 2000; Zollner et al., 2000; Menzies, 2003; Prévost et al., 2003; Rainard et al., 2003; Arrecubieta et al., 2006; Diep et al., 2006; Zecconi et al., 2006; Reinoso et al., 2008; Zhao & Lacasse, 2008; Cremieux et al., 2009; Gogoi-Tiwari et al., 2015; Ahangari et al., 2017) are defined in (Table 1). At the starting of the infection, *S. aureus* has various surface proteins, called “microbial surface components identifying adhesive matrix molecules (MSCRAMMs),” facilitating adherence to the host tissues. “MSCRAMMs bind molecules” for example, collagen, fibrinogen, and fibronectin, as well as others, may stick to the similar components of the host-tissue. *S. aureus* can propagate and persevere in various ways once adheres to host tissues or prosthetic materials. *S. aureus* has several other features that playing an essential role in evading host immunity during infection; for example, producing an anti-phagocytic microcapsule and abscess formation by the zwitterionic capsule (O’Riordan & Lee, 2004; Foster, 2005). *S. aureus* may also prevent neutrophil migration and chemotaxis releasing to the location of infection due to it can secrete the *Staphylococci* inhibitory protein or the extracellular adherent protein (Stephan et al., 2001).

**Table 1.** Selected *Staphylococcus aureus* virulence factors.

Virulence factors which have a key role in:	Selected factors	Genes	Form of mastitis	References
Attachment	MSCRAMMs such as “clumping factors, fibronectin-binding proteins, collagen, and bone sialoprotein-binding proteins.”	clfA, clfB, fnbA, fnbB, cna, fib, bbp	Clinical, subclinical	(Matsunaga et al., 1993; Menzies, 2003; Reinoso et al., 2008; Ahangari et al., 2017)
Persistence	Biofilm accumulation such as “polysaccharide intercellular adhesion, small-colony variants, and intracellular persistence”	ica locus, hemB mutation	Subclinical, recurrent chronic	(Zhou et al., 2018,Arrecubieta et al., 2006)
Attacking and destroying host immune system	Leukocidins such as “PVL and g-toxin, capsular polysaccharides, protein A, CHIPS, Eap, and Phenol-soluble modulins.”	lukS-PV, lukF-PV, hlg, cap5 and 8 gene clusters, spa, chp, eap, psm-a gene cluster	Clinical < Subclinical	(Rainard et al., 2003; Arrecubieta et al., 2006; Cremieux et al., 2009; Gogoi-Tiwari et al., 2015)
Invasion and penetration of tissue	“Nucleases, hyaluronate lyase, phospholipase C, and metalloproteases (elastase), Proteases, lipases.”	V8, hysA, hla, plc, sepA	Clinical	(Dinges et al., 2000; Zhao & Lacasse, 2008)
Toxin-mediated-disease and/or sepsis	“Enterotoxins, toxic shock syndrome toxin-1, exfoliative toxins A and B, a-toxin, peptidoglycan, and lipoteichoic acid.”	sea-q (no sef), tstH, eta, etb, hla	Peracute < acute < chronic	(Matsunaga et al., 1993; Prévost et al., 2003; Zollner et al., 2000; Zecconi et al., 2006)
With a poorly definite role in virulence	“Coagulase, ACME, and a bacteriocin.”	arc cluster, opp-3 cluster, bsa	Peracute, acute, chronic	(Matsunaga et al., 1993; Diep et al., 2006)

*Note*. ACME: arginine catabolic mobile element; CA-MRSA: community-acquired methicillin-resistant *S. aureus*; CHIPS: chemotaxis inhibitory protein of *Staphylococci*; Eap: extracellular adherence protein; MSCRAMMs: microbial surface components recognizing adhesive matrix molecules; PVL: Panton-Valentine leukocidin.

**Figure 2.**
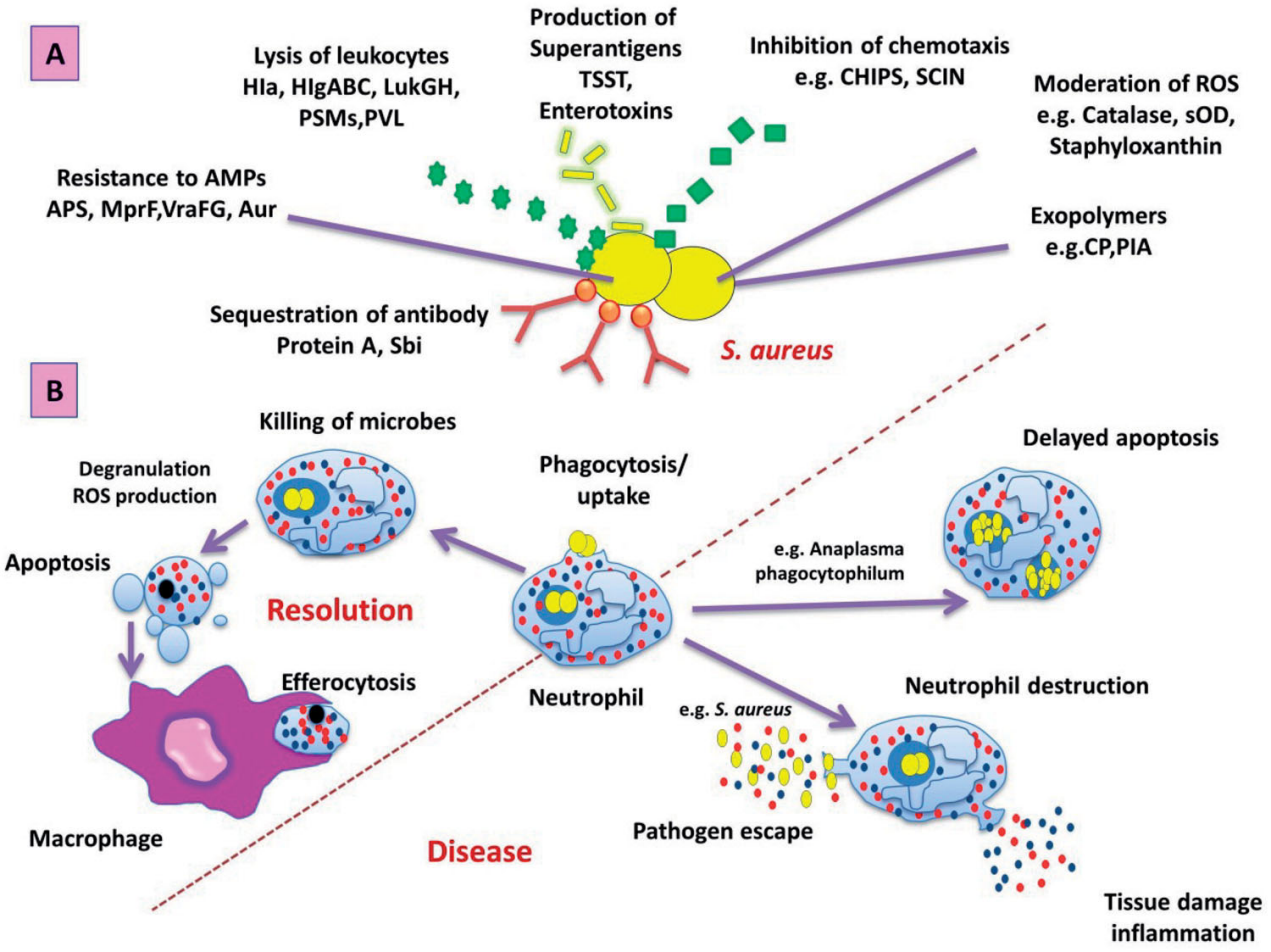
Mechanisms of the immune system against *S. aureus* infection. (A) *S. aureus* attacks the immune system by various trials as preventing identification, preventing chemotaxis, regulating ROS, Resistance to Amp, and directly lysis of leukocytes. (B) Phagocytosis of bacteria by neutrophil leads to increased ROS and degranulation, which help in killing the ingested microorganism and resulted in apoptosis of neutrophil that can be removed by macrophage to aid in the resolution of infection (Rigby & DeLeo, 2012). Alternatively, bacteria may change in normal neutrophil by accelerating a delay of apoptosis or enhanced neutrophil damage, escaping the pathogen into the tissue and the occurrence of disease (Coxon et al., 1996). Abbreviation: APS: antimicrobial peptide-sensing system; Aur: aureolysin; CHIPS: chemotaxis inhibitory protein of *S. aureus*; CP: capsular polysaccharide; Hla: α-toxin; HlgABC: γ-hemolysin; LukGH: LukF-G and Luks-H; MprF: multiple peptide resistance factor; PIA: polysaccharide intercellular adhesion; PSMs: Phenol-soluble modulins; PVL: Panton-Valentine leukocidin; Sbi: second binding protein of immunoglobulin; SCIN: staphylococcal inhibitor of complement; SOD: superoxide dismutase; VraFG: vancomycin resistant-associated gene.

Additionally, *S. aureus* produces leukocidins responsible for leukocyte destruction by the creation of apertures in the cell membrane. During infection, *S. aureus* produces various enzymes, like (proteases, lipases, and elastases) that alter it to attack, damage host tissues and unfold to alternative places (Figure 2).

**Figure 3.**
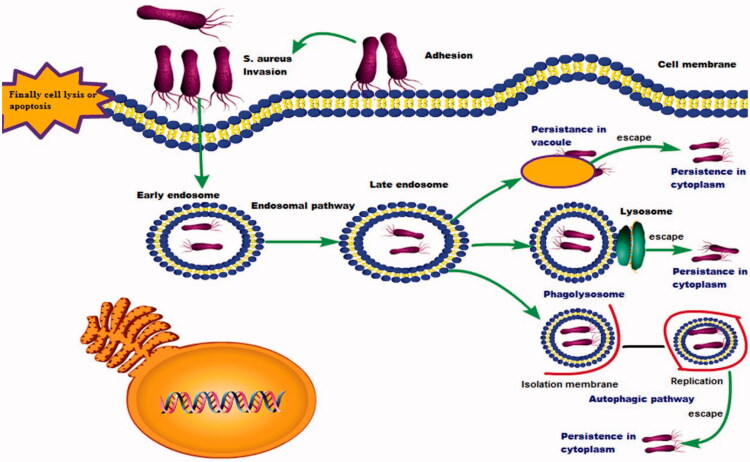
The intracellular parasitism of the *S. aureus*.

The intracellular persistence of the *S. aureus* in the macrophages and mammary epithelium, leading to more difficulty in its treatment. As well as, the *S. aureus* has the ability to live and reside in special cell compartments as the endosome and the cytosol, leading to considerable obstacles in their cleaning from the body and establish a reservoir from which the repeated infection will occur as shown in (Figure 3).

Besides, the small-colony variants (SCVs), are another form of *S. aureus* contributing to persistent and repeated infection. *In vitro*, SCVs are competent to “hide” in host cells without causing a significant damage effect and are relatively protected from antibiotics and host defense mechanisms after that; they can relapse to the more infectious wild-type phenotype, probably causing recurrent infection (Zhou et al., 2018).

**Figure 4.**
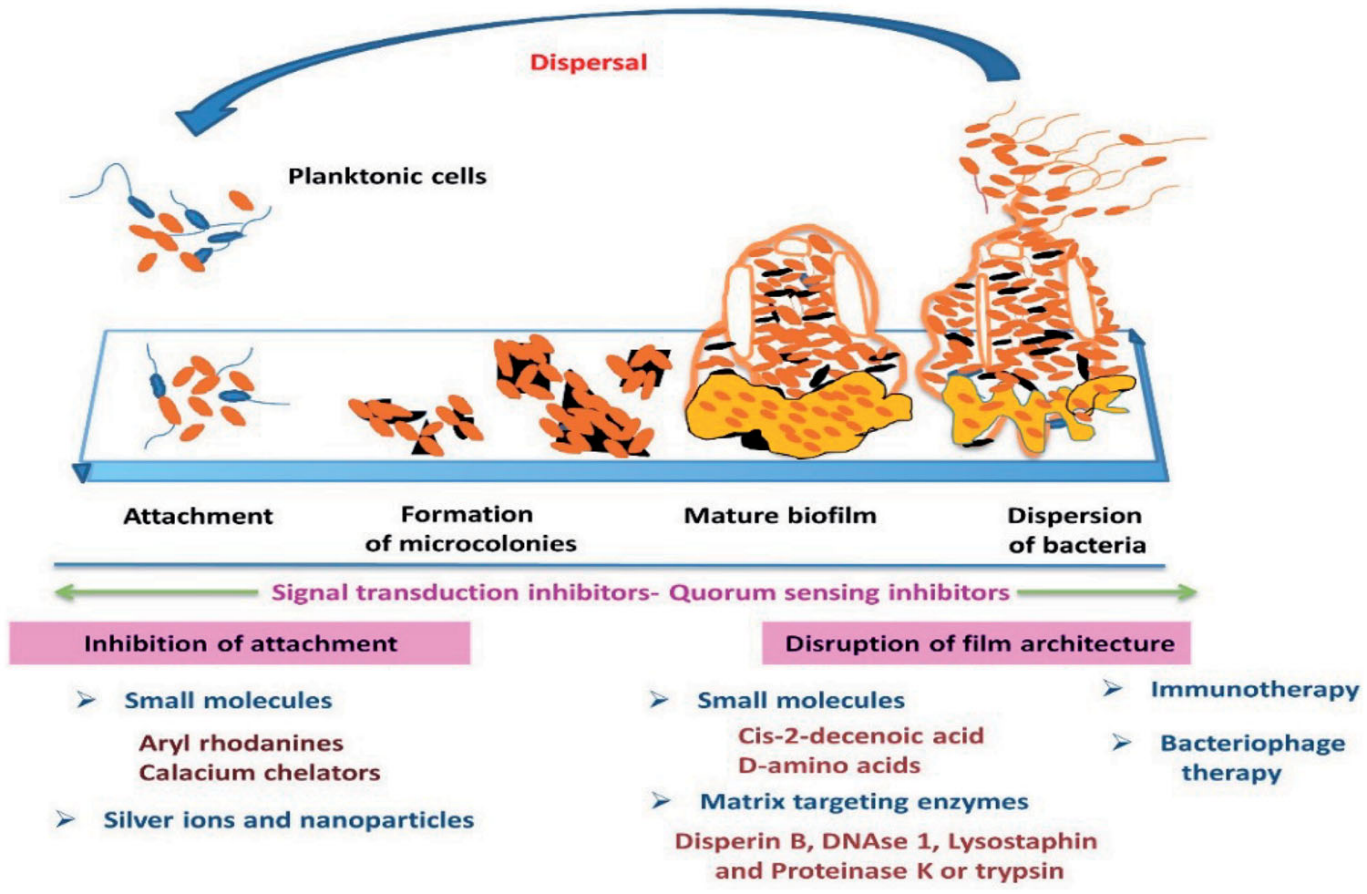
Strategies in the development and management of the biofilms.

Perseverance of *S. aureus* by attacking host battlements and antimicrobials, owing to its ability to produce biofilms “slime” on prosthetic surfaces and a host (Dinges et al., 2000). If the prosthetic device is infected, for example, it will be difficult to reduce the infection without device removal (Arrecubieta et al., 2006). *S. aureus* has the ability to create biofilm by four stages as shown in (Figure 4) and it acts as an imperative virulence feature and associated with various syndromes including mastitis owing to its ability to persuade persistent antimicrobial resistance (Thurlow et al., 2011), delay phagocytosis, and either reduce or encourage inflammation, according to the disease pattern (Fernandes et al., 2011; Atulya et al., 2014).

Biofilm formation is a dynamic process, rendering the possible detaching of planktonic cells that rapidly multiplied and inhabited on other surfaces. This process has a highly significant effect on promoting the presence of a microbial pathogen in other infection locations, and subsequently, created new biofilms and wide spreading of the infections. Lately, it was proved that immune responses against experimentally induced acute mastitis in mice by *S. aureus* in biofilm form were stronger than planktonic cultures (Gogoi-Tiwari et al., 2015). Repetition the symptoms of infection are occurred due to a modification in the toxin and adhesion molecules gene expression, as well as a fast multiplication that followed the process of detachment. Numerous types of genes are intricated in biofilm development, for instance, (loci ica) intercellular adhesion (Vasudevan et al., 2003), (bap) biofilm-associated protein, (agr) accessory-gene regulator and (sar) staphylococcal-accessory regulator (Cucarella et al., 2004; Planchon et al., 2006; Gomes & Henriques, 2016). Therefore, the potential function of biofilms in chronic infections drew the attention of scientists in the description of biofilm development (Tormo et al., 2005).

Regardless of the clinical manifestation of mastitis, acute or sub-acute and/or chronic, the development of biofilm by *S. aureus* has been planned to happen in two steps (Boonyayatra et al., 2014), the first step is occurred by the action of (MSCRAMMs) for helping the attachment of *S. aureus* with the mammary gland epithelial cells. Where *S. aureus* with the biofilm-forming ability is attached firmly to the epithelium of the udder more than the non-biofilm form (Brouillette & Malouin, 2005). Secondly, the attached *S. aureus* is multiplied and accumulated by the bacterial extracellular matrix involving, (polysaccharide intercellular adhesion (PIA)) produced by (icaADBC) that is considered the most virulent factor associated with biofilm creation (Otto, 2013).

However, ica-independent biofilm creation has similarly been recorded in a small percent (Gogoi-Tiwari et al., 2015). Furthermore, cytolytic toxins produced by *S. aureus* have also been stated to be vital in biofilm establishment (Huseby et al., 2010). Alpha-toxin is very important in cell communications (Caiazza & O’Toole, 2003), while beta-toxin forms an insoluble nucleoprotein matrix in the presence of deoxyribonucleic acid (DNA) via covalent cross-links (Huseby et al., 2010). Additionally, the epithelium cells lining the teats and gland cisterns of the quarter can be destructed by alpha (α) and beta (β) toxins consequentially, leading to direct damage to the milk-producing tissue, creation deep-seated cavities of infection in the alveoli, and presence of scar tissue in cases of acute mastitis (Petersson-Wolfe & Jones, 2010). Protein A, and biofilm-associated protein (Bap), which included in biofilm formation, have a role in perseverance intracellular and antibacterial resistance (Cucarella et al., 2004; Valle et al., 2012). Also, up and down-regulation of agr activity play the primary function in biofilm development whereas the low activity is essential for biofilm creation by up-regulation of bacterial surface components or adhesins; however, the spreading of biofilm is controlled by the secretion of proteases and nucleases, which are inspired by agr activation (Boonyayatra et al., 2014). Therefore, biofilm creation is a noteworthy supplier to *S. aureus* pathogenesis, and the necessity for substitute treatments that directly challenge this element is of most importance.

## 3. Advantages of nanoparticles in the treatment of *S. aureus mastitis*

The failure of *S. aureus* therapy is occurred due to: its ability for intracellular persistence within the phagocytes and due to the antimicrobial resistance. This may be caused by reducing the uptake rate of usually-used antibiotics intracellular or to their action and activity that were decreased at the acidic pH of lysosomes; the non-dispersion of acidic drugs through the lysosomal membrane attributable to their ionic appearance at neutral extracellular or cytoplasmic pH, and decreasing the retention rate of antibiotics in cells. For all these causes, when antibacterial drugs are used in aqueous solutions, its activity is not still present continuously. Therefore, there are absolute needs for more particular dosage forms to be valid in the cure of *S. aureus* infection and, if possible, should have these merits: (1) penetration of phagocytes to vast scope and reserve in cells for a suitable time; (2) retaining no or low metabolism in the cells; (3) revealing stronger activity at acidic pH against *Staphylococci*; and (4) administration through the streak canal. Nanoparticles are anticipated new dosage form to be used intramammary to obtain the effective effects.

Nanoparticles drug delivery systems have different functional and biological properties (Garg et al., 2015b; 2015c). They easily to be modified by changing the dose and the ratio of the drug, the materials which enter in their synthesis as the polymer, the excipient, the stabilizer, and others to solve the problems which accompanied by the conventional medication (Garg et al., 2015d).

### 3.1. Enhanced antibacterial activity against resistant *S. aureus*

In recent years, some studies have been performed the development of highly targeted nanomaterials to overcome the antibiotic-resistant bacteria (Baptista et al., 2018). These nanomaterials have the ability to incorporate one or more drugs without any effect on the structure of the cargo and increase the pharmacological action of the payload (Pissuwan et al., 2011; Gholipourmalekabadi et al., 2017). Due to they have many merits as uniform dosing of the drug, increase its bioavailability, deliver the drug at the infected site, decrease the therapeutic timing and side effects, additionally, prevent burst release and degradation of the drug (Garg et al., 2015d).

**Figure 5.**
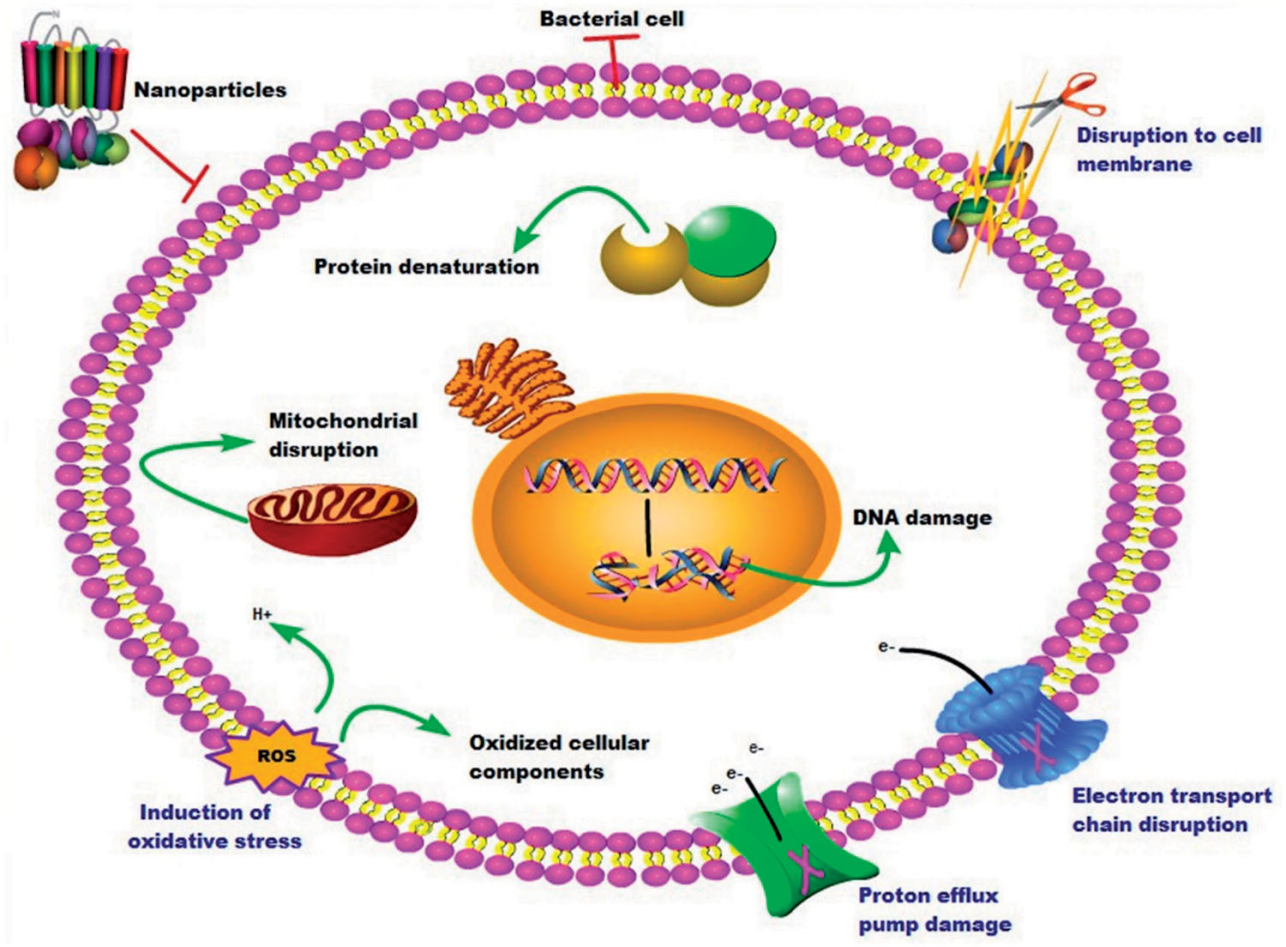
The cytotoxic effect of the nanoparticles on bacterial cells.

Besides the importance of the nanomaterials in protection of the drug from degradation and delivery to the infected site, nanomaterials themselves can be cytotoxic and destructive to the bacteria by different mechanisms. The nanoparticles interact with the bacterial cell membrane and leading to its destruction, generating reactive oxygen species, enzymatic inactivation, protein deactivation, changes in the gene expression, and they stimulate innate and adaptive immunity (Wang et al., 2017) (Figure 5). These bactericidal pathways help the nanomaterials to overcome the antibiotic-resistant mechanism. Esmaeillou et al. (2017), demonstrated that the silver nanoparticles could overcome the vancomycin-resistant in cases of *S. aureus* through binding with the vancomycin and enhancing bacterial cell death. Also, Saeb et al. (2014), reported that silver nanoparticles could avoid the methicillin-resistant in cases of *S. aureus* through binding with the antibiotic. The gold nanoparticles enhanced the antibiotic activity of ampicillin in ampicillin-resistant *S. aureus* by binding with ampicillin and entry it to the bacterial cell (Brown et al., 2012).

### 3.2. Inhibition of biofilm formation

As well as, the nanomaterials have a significant impact on the treatment of *S. aureus* infection by preventing the biofilm formation whereas, the main component of the biofilm is the glycocalyx with anionic charge, it can interact with the nanoparticles with a positive charge which have the ability to penetrate the thick biofilm (Kulshrestha et al., 2017). Sathyanarayanan et al. (2013), reported that the gold nanoparticles had a significant decrease in the biofilm that was formed by *S. aureus*. Liu (Liu, 2019), mentioned that using triclosan as an antimicrobial drug in solution only penetrate and killing the *S. aureus* outside the biofilm; however, loading the triclosan in micellar nanocarrier help in the penetration of staphylococcal biofilm and killing the bacteria over the depth of the biofilm. Also, some authors demonstrated that inhibition of the biofilm could be achieved by interfering with the quorum-sensing systems (QSs), which act as a major regulatory mechanism in biofilm formation (Figure 4) (Singh et al., 2017). Modifying the nanoparticle surface by some substance such as “B-cyclodextrin or N-acylated homoserine lactonase proteins” can switch off the (QSs) and prevent the bacterial communication through interfering with the signal/receptor interaction (Kato et al., 2006; Ortíz et al., 2008).

### 3.3. Enhanced intracellular delivery

The nanoparticles penetrate the cell membrane, and subcellular organelles then deposited in the infected site by different pathways. These pathways have been discussed in detail in our previous works (Xie et al., 2014; Zhou et al., 2018). The various transport ways affect on the drug uptake, distribution between the cells, and its therapeutic action. The nanoparticles remain intracellular for a long time and release their cargo through the pores which present in the nanoparticle’s membranes and their response to the external stimuli as the changes in the PH, temperature, redox, and others. Therefore, during preparation of the nanoparticles, we need to synthesis on-demand release nanoparticles to release the payload drug in the right site. Most of the nanoparticles release their cargo in the endosome and the lysosome according to the nature of the nanomaterials, so in the cases of the *S. aureus* mastitis, we need to use nanoparticles have the ability to deliver the drug to the endosome then release it to the cytosol. We can achieve it by preventing the degradation of the drug in the lysosome through the “proton sponge” effect by ejecting the drug from the late endosome through disruption the endosomal compartment by using polycations as polyhistidine and poly amino esters which binds with the endosomal membrane and promote osmotic swelling of the endosome then destabilization and disruption the membrane. Therefore, the researchers are working on designing and developing of nanoparticles drug delivery system to improve the therapeutic action of the payload drug and decrease the toxic side-effect. It could be achieved by making different formula and choose the best optimum method through changing in various variable factors as the polymer/cross-linker ratio, stirring power, sonication time, cross-linking time to form the final product with specific criteria (Cui et al., 2006; Graf et al., 2009).

**Figure 6.**
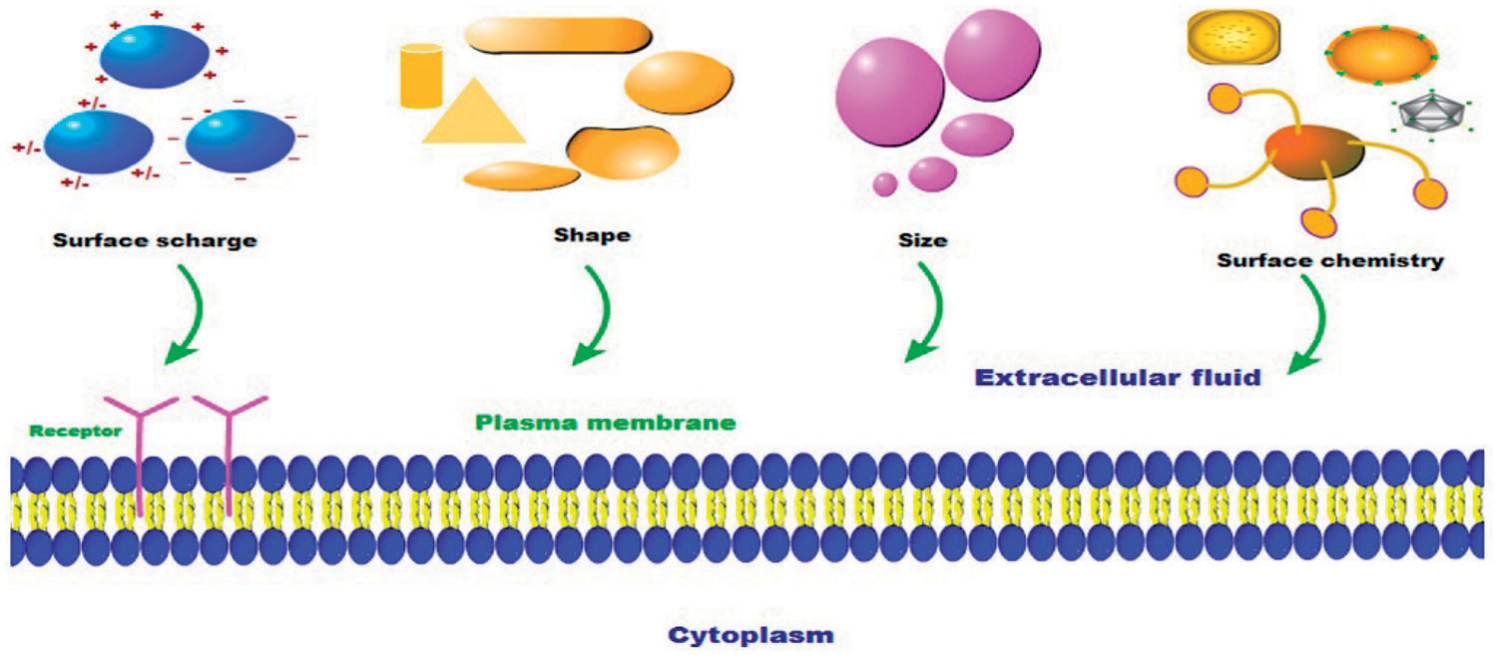
Physicochemical parameters of nanoparticles that influence on their payloads.

The intracellular delivery of the nanoparticles affected by their physicochemical parameters **(**Figure 6**)** such as the nanoparticles size, shape, and synthetic chemistry:

The size of the nanoparticles acts as a key factor in the intracellular uptake of the nanoparticles and the behavior of them in the biological fluid. Also, it detects the *in vivo* biodistribution of the nanoparticles, their stability, the drug loading and release, and the nanoparticles toxicity. Many studies showed that the submicron size has advantages than the micron size; therefore, the small particles are more effective than the large particles in the drug delivery to the infected loci. For example, Yuan et al. (2017), demonstrated that the silver nanoparticles with small sizes range from 10 nm to 50 nm were effective in the treatment of the *S. aureus* causing mastitis in goat. Chithrani et al. (2006), mentioned that the silver nanoparticles with size 50 nm showed the maximum cellular uptake in mammalian cells, and the adipose-derived stem cells (Ko et al., 2015). Therefore, it is a promising way to deliver the drug intracellular in the special *S. aureus* compartments via controlling the size of the nanoparticles.

The shape of the nanoparticles also affects on their cellular uptake, biological behavior, macrophage phagocytosis, and the pharmacokinetics of its payload drug. There are different shapes from the nanoparticles (spherical, rod, ellipsoid, etc.). Shi et al. (2016), designed a spherical shape nanoparticle with 15–25 nm from the chitosan and loaded with the iron oxide to penetrate the thick biofilm formed by the *S. aureus*. Maya et al. (2012), showed that the rod shape chitosan nanoparticle loaded with tetracycline had the ability to be uptaken by the macrophage and the epithelial cells and killing the intracellular *S. aureus* whereas it killed the intracellular *S. aureus* more six-fold than the tetracycline alone by increasing concentration of the tetracycline in the infected site. Kalhapure et al. (2014), demonstrated that the spherical shape of the linoleic acid solid lipid nanoparticles loaded with vancomycin with sizes range from 95 to 100 nm showed an effective treatment for the sensitive and the resistant form of *S. aureus* infection. Therefore, the nanoparticles can be designed with unique shapes to control their cellular entrance and their functions intracellular.

The surface chemistry of the nanoparticles plays a vital role in detecting their behavior in the biological fluid due to they are affected by type of the biomolecules attached to the surface of the nanoparticles and their composition. The nanoparticle surface chemistry is not constant due to attachment and detachment of the biomolecules according to their binding affinity to the surface. There are stabilizing molecules as the PEG, albumin, DNA, and others can be used to decrease the ionic strength and prevent nanoparticle aggregation and agglomeration (Ehrenberg et al., 2009; Gillich et al., 2013).

The change in the nanoparticle charge affects on the pattern of the endocytosis process; for example, cellular uptake of the polymeric nanoparticles differs by using positive charge particles as (chitosan hydrochloride) and negative charge particle as (carboxymethyl chitosan). The phagocytic uptake of the positive charge polymeric nanoparticles is higher than the negative and the neutral charge nanoparticles (He et al., 2010). However, reaching of the positive charge nanoparticles to the infected site is considered difficult owing to their nonspecific binding to the normal tissue (Kim et al., 2010), and the negative charge nanoparticles may be better when it delivers the drug deep into the tissue (Kim et al., 2010). Therefore, to take the benefits from the two opposite charges, we can design type from the nanoparticles to carry a negative charge in the healthy tissue and positive charge in the acidic inflamed tissue (Miao et al., 2018).

Moreover, the functionalization of the nanoparticle surface with “PEG, poloxamer, poloxamine polymers, and other” prevent their phagocytosis because these polymers decrease the nanoparticle ionic strength, their aggregation, and the absorption of the protein which present on their surface, also enhance their dispersion (Moghimi, 1999).

### 3.4. Enhanced the activity against the small-colony variants (SCVs) of *S. aureus*

SCVs are one significant reason why *S. aureus* infections remain clinically challenging due to it is difficult to be eradicated by the antimicrobial drug and the immune system. Therefore, some researchers are trying to improve the activity of antibacterial drugs against SCV phenotypes of *S. aureus* by using nanoparticles. Subramaniam et al. (2019), used the mesoporous silica nanoparticles (MSNP) loaded with the rifampicin as a nanocarrier system in the treatment of intracellular infection caused by SCVs of *S. aureus*. In this study, the MSNP with two sizes 40 nm and 100 nm loaded with the rifampicin showed more bactericidal activity than rifampicin alone by enhancing the intracellular rifampicin uptake by RAW 264.7 macrophage infected with SCVs of *S. aureus*. Also, Heck et al. (2018) used Zirconyl Clindamycin phosphate inorganic-organic hybrid nanoparticles as a novel nanocarrier to deliver the drug, and they observed that this type of nanocarrier delivered high concentration from the drug (70–150) times than the free drug to the infected site of the SCVs *S. aureus*.

## 4. The current progress of different nanoparticle delivery systems for enhancing *S. aureus* infection therapy

Several researchers confirmed that different nanoparticles, including organic and inorganic nanoparticles, could have likely been used in medical research, specifically for bovine mastitis infections. There are various mechanisms by which nanoparticles have the potency to inhibit the antimicrobial resistance by killing the bacteria, improving the performance of existing antibiotics via keeping them from detection, degradation, and providing a means of targeted delivery to the microorganisms to utilize the lowest concentration from drugs (Wong et al., 2013). Additionally, hindering the bacterial adhesion, colonization, and biofilm development.

Currently, some nanocarriers can be utilized to promote the pharmacological activities against sensitive and resistant *S. aureus* through conjugated or incorporated with many antimicrobial agents. So, nanoparticle delivery for drugs considered an ideal tool to overwhelmed the *S. aureus* infection. The permeability of drugs within cells can be boosted by the nanoparticles. Nanoparticles can enhance the antibacterial activity of antibiotics against intracellular *S. aureus* infection by increasing the diffusion of released drugs to the main target, increasing its uptake by cells, and improving the release of the nanoparticle entrapped or adsorbed antibiotics. The chemical compound nanoparticles with ionic core and specific hydrophobic/hydrophilicity chemistry of the shell can also produce proficient action against microorganism through binding with plasma membrane by a hydrophobic phase of the shell, and stronger fixed interacted with the alternative surface charge of the core. The phagocytosis efficiency can be developed by adapting the nanoparticles with specific ligands of macrophage for enhancing the intracellular concentration of antimicrobial agents (Hua et al., 2014). The main nanoparticles, being researched for *S. aureus* infection are liposomes, polymeric nanoparticles, solid lipid nanoparticles, nanogels, and metal nanoparticles. Therefore, the next section will introduce the progress, advantages, and disadvantages of each type from these nanoparticles in the therapy of *S. aureus* infection.

### 4.1. Liposomes

Liposomes are considered as spherical vesicles involving of at least one amphiphilic lipid bilayer with an internal aqueous core being them just resembling a cell membrane. The lipid bilayer can be extra amplified with additional components, such as cholesterol or polyethylene glycol (PEG), with the intention of progress stability or biological withholding (Pattni et al., 2015). The drug can be loaded into liposomes for enhancing successful therapy (Table 2). For example, ciprofloxacin was overloaded into liposomes with 45% loading efficiency; as well as, adding cysteine to dithiobenzyl urethane linkage in middle of the lipid and the PEG, increasing release level of the encapsulated ciprofloxacin (Karathanasis et al., 2005). Liposomes also considered a probable carrier for the intracellular distribution of antibacterial drugs attributable to their phospholipid bilayer structure. Thus, for example, the activity of anti-biofilm and antibacterial agents can be augmented by levofloxacin liposomes (Gupta et al., 2017) and ceftazidime liposomes (Zhou et al., 2012). As similar, chloramphenicol-loaded deoxycholic acid liposomes have antimicrobial action against MRSA (Hsu et al., 2017), and vancomycin-loaded liposomes increase and enhance the intracellular killing of MRSA (Pumerantz et al., 2011). Large unilamellar liposomes can be used as a carrier to streptomycin for combating the intracellular infection of *S. aureus* (Bonventre & Gregoriadis, 1978). A study reported that gentamycin load liposomes could inhibit the activity of *S. aureus* than free gentamycin (Dees & Schultz, 1990). Liu et al. (2016), said that azithromycin-loaded liposomes showed higher bactericidal action against MRSA as the result that reported by Li et al. (2015) who used optimum ratio from clarithromycin and daptomycin-loaded liposomes. Jijie et al. (2017) mentioned that more than antibiotics could be entrapped as piperacillin and a β-lactam into the liposomes to increase the antibacterial action against *S. aureus*. Also, Nigatu et al. (2018) discussed that liposomes could be modified to be sensitive to the higher temperature of the inflamed site at 39 °C and release the loaded drug at the target site. The liposomes decrease the drug toxicity, increase the pharmacological action of the drug by change in its pharmacokinetics and biodistribution, and they are safe for parental administration, but its stability is limited due to decrease the shelf lives of the lipid vesicles, and it is complicated in its preparation and expensive (Allen & Martin, 2004; Gabizon et al., 2006).

**Table 2.** Summary of the most recent examples of nanoparticles for improving the antibacterial delivery against *S. aureus* infection.

No.	Nanocarriers	Antibacterial drugs	Method of the preparation	Size	Route of administration	Performance	References
1	Liposomes	Levofloxacin	…………	200–300 nm	In vivo I/P In vitro	Inhibited biofilm formation	(Gupta et al., 2017)
2	Liposomes	Ceftazidime	Modified reverse-phase evaporation method	161.5 ± 5.37 nm	In vitro	Inhibited biofilm formation	(Zhou et al., 2012)
3	Deoxycholic acid liposomes	Chloramphenicol	…………	239 nm	In vivo S/C In vitro	Enhanced antibacterial effect against MRSA.	(Hsu et al., 2017)
4	Liposomes	Vancomycin	A hydration– dehydration method	254 ± 147 nm	In vitro	Enhanced bactericidal effect against intracellular MRSA.	(Pumerantz et al., 2011)
5	Liposomes	Streptomycin	…………	…………	In vitro	Enhanced bactericidal effect against intracellular *S. aureus*.	(Bonventre & Gregoriadis, 1978)
6	Liposomes	Gentamycin	…………	…………	In vitro	Increased intracellular accumulation and subcellular distribution of the drug. Improved antibacterial effect against *S. aureus*.	(Dees & Schultz, 1990)
7	Liposomes	Azithromycin	Film dispersion method,	100 nm	In vitro In vivo	Enhanced antibacterial effect against MRSA.	(Liu et al., 2016)
8	Liposomes	Daptomycin	…………	98.2 ± 2.21 nm	In vitro In vivo	Enhanced antibacterial effect against MRSA.	(Li et al., 2015)
9	Liposomes	Piperacillin and a β-lactam	…………	…………	In vitro	Enhanced antibacterial effect against *S. aureus*.	(Jijie et al., 2017)
10	Chitosan	Iron oxide nanoparticles	…………	15–25 nm	In vitro	Inhibited biofilm formation.	(Asli et al., 2017)
11	Chitosan	Cloxacillin			In vitro	Inhibited biofilm formation.	(Breser et al., 2018)
12	Folic acid tagged chitosan	Vancomycin	Coavalent linkage method	260 ± 35 nm	In vitro	Enhanced bacterial efficacy against VRSA.	(Chakraborty et al., 2010)
13	Chitosan	Tetracycline	Ionic crosslinking method	200 ± 20 nm	In vitro	Inhibited intracellular infection of *S. aureus*	(Maya et al., 2012)
14	Chitosan	Bacillus natto			In vitro	Inhibited biofilm formation	(Jiang et al., 2017)
15	PLGA	Gentamycin	…………	…………	In vitro	Enhanced antibacterial efficacy of gentamycin	(Imbuluzqueta et al., 2010)
16	PLGA	Ciprofloxacin	Double emulsion solvent evaporation method	300 nm	In vitro	Inhibited biofilm formation	(Thomas et al., 2016)
17	Cap-PLGA	Nafcillin sodium, levofloxacin	Emulsion solvent evaporation technique	…………	In vitro	Inhibited biofilm formation	(Bastari et al., 2014)
18	Polyacrylate	Ciprofloxacin	Emulsion polymerization	40 nm	In vitro	Increased therapeutic efficacy against *S. aureus*.	(Turos et al., 2007)
19	SLNs	Tilmicosin	Hot homogenization and ultrasonication method	343 ± 26 nm	In vitro In vivo S/C	Better therapy to *S. aureus* mastitis.	(Wang et al., 2012)
20	HCO-SLNs	Tilmicosin	O/W emulsion–solvent evaporation technique	90–230 nm	Invivo S/C Invitro (COS-7 and MDBK cells)	As a good carrier for tilmicosin controlled and sustained release in cases of *S. aureus* mastitis.	(Han et al., 2009)
21	Dosonic acid-SLNs	Enrofloxacin	Hot homogenization and ultrasonication method	150.1 nm–605.0 nm	In vitro RAW 264.7 cells	Enhanced antibacterial action by increased intracellular accumulation of the drug.	(Xie et al., 2017)
22	SLNs	Vancomycin	Hot homogenization and ultrasonication method	102.7 ± 1.01 nm	In vitro	Anti-MRSA effect.	(Kalhapure et al., 2014)
23	SLNs	Florfenicol	Hot homogenization and ultrasonication method	253 ± 3 nm	In vitro In vivo	Enhanced the therapeutic efficacy of the drug.	(Wang et al., 2015)
24	SLNs	Retinoic, Lauric acid	…………		In vitro	Enhanced the therapeutic efficacy of the drug against *S. aureus*.	(Silva et al., 2015)
25	Nanogel	Copper	…………	50 nm	In vitro Ex vivo	Used in cases of clinical mastitis.	(Krishna et al., 2017)
26	RBCs-nanogel	Vancomycin	Cross-linking method	100.8 ± 0.3	In vitro	Enhanced antivirulence and antibacterial effect against MRSA. Toxin neutralization.	(Zhang et al., 2017)
27	RBCs-hydrogel	PLGA nanoparticles	…………	…………	In vivo	Toxin neutralization of *S. aureus*.	(Wang et al., 2015)
28	Dextran-nanogel	Zinc nitrate	Polymerization and crosslinking by inverse miniemulsion	250 nm	In vitro	Anti-MRSA action.	(Malzahn et al., 2014)
29	Dextran-lysosyme nanogel	Silver nanoparticles	…………	5 nm	…………	Enhanced antibacterial action against *S. aureus*.	(Ferrer et al., 2014)
30	Carbapol Aqua SF1	Vancomycin	Swelling-deswelling mechanism	400 nm	In vitro	Enhanced bactericidal effect against *S. aureus*.	(Mohammed et al., 2018)
31	Poly-N-Iso Propyl acrylamide-nanogel	Silver Nanoparticles	Insitu reduction method	135 nm–532 nm	In vitro	Enhanced bactericidal effect against *S. aureus*. Decreased aggregation of silver nanoparticles.	(Qasim et al., 2018)
32	Poly acryclic acid	Silver nanoparticles	Electron beam irradiation	Around 200 nm according to the irradiation dose	In vitro In vivo	Good bactericidal effect against *S. aureus*.	(Choi et al., 2013)
33	Alginate nanocomposite hydrogel	Silver nanoparticles	…………	…………	In vitro	Enhanced bactericidal effect against *S. aureus*.	(Stojkovska et al., 2014)
34	Acrylamido-methylpropane sodium salt hydrogel	Silver nanoparticles	Ultraviolet radiation	…………	In vitro	The greatest inhibitory effect against MRSA	(Boonkaew et al., 2014)
35	Fumaric acid cross linked-carboxy methyl acetate hydrogel	Silver nanoparticles	Cross linking method	…………	In vitro	Inhibited *S. aureus* infection by 99.99%	(Bozaci et al., 2015)

*Note.* MRSA: methicillin-resistant *Staph. aureus*; VRSA: vancomycin-resistant *Staph. aureus*; PLGA: poly-lactide co glycolide; Cap: calcium phosphate; SLNs: solid lipid nanoparticles; RBCs: red blood cells.

### 4.2. Polymeric nanoparticles

Polymeric nanoparticles are one of the greatest nanoparticle therapeutics used in research, which have been generally explored as a promising platform for antibiotic delivery (Table 2). Polymeric nanoparticles are made-up from (biocompatible and biodegradable) polymers and are expressed by a self-assembly process using block-copolymers, including two or more polymer chains with a lot of hydrophilicity. Furthermore, polymeric nanoparticles have been prepared to enclose either hydrophilic or hydrophobic drug molecules, macromolecules as nucleic acids, proteins, and peptides (Wang et al., 2012). Chitosan acts as a drug delivery carrier due it has several benefits, for instance, biocompatibility, biodegradability, nontoxic, and inexpensive. Asli et al. (2017), mentioned that chitosan molecules prevent biofilm created by *S. aureus* isolates in bovine mastitis. Furthermore, Shi et al. (2016), demonstrated that chitosan-coated iron oxide compound nanoparticles are hindering the development of biofilm biomass and declining the quantity of live bacterium. A study reported by Breser et al. (2018) showed that the combination between chitosan and cloxacillin combination inhibited biofilm formation and reduced intracellular viability of coagulase-negative *Staphylococcus* in cases of chronic mastitis. Chakraborty et al. (2010) discussed that vancomycin loaded folic acid tagged chitosan nanoparticles had a higher bactericidal effect against vancomycin-resistant *S. aureus* by enhancing the transport of vancomycin across bacterial cell membranes. Intracellular infection by *S. aureus* was also inhibited by tetracycline loaded chitosan nanoparticles (Maya et al., 2012), and biofilm formation was inhibited by Bacillus natto loaded chitosan nanoparticles (Jiang et al., 2017). PLGA (poly-lactic-co-glycolic acid) nanoparticles also act as a carrier to antibacterial agents whereas gentamycin loaded PLGA nanoparticles exhibited higher antibacterial effect against *S. aureus* through increased intracellular accumulation and distribution of gentamycin (Imbuluzqueta et al., 2010). Thomas et al. (2016), reported that biofilm formation of *S. aureus* was inhibited by ciprofloxacin-loaded PLGA nanoparticles and by nafcillin sodium and levofloxacin-loaded calcium phosphate PLGA nanoparticles (Bastari et al., 2014). Moreover, Turos et al. (2007), showed that glycosylated polyacrylate nanoparticles enhanced the therapeutic efficacy of ciprofloxacin against *S. aureus* infection. The polymeric nanocarriers have more advantages, including increased drug bioavailability and encapsulation efficiency, released the payload in a controlled behavior, and concentrated the drug release in the inflammatory and the infected site (Kumari et al., 2010). However, presence of the reactive group may influence the rate of the conjugation reaction and the stability of the polymer (Jijie et al., 2017).

### 4.3. Solid lipid nanoparticles

Solid lipid nanoparticles (SLNs), which considered an alternative drug carrier to the polymeric and liposome nanoparticles, attracted the attention because of their biocompatibility, biodegradability, stability (Xie et al., 2011), and accordingly, might be a promising carrier for a drug that is used to treat intracellular infections (Xie et al., 2014). For example, Wang et al. (2012), and Han et al. (2009), demonstrated the potential effect of tilmicosin-loaded SLNs against *S. aureus* mastitis. Also, our previous work (Xie et al., 2017; Li et al., 2019) indicated that enrofloxacin-loaded docosanoic acid SLNs could successfully increase the accumulation and storage time of enrofloxacin within the cell and we recently improved the palatability, stability and oral bioavailability of enrofloxacin by an enteric coating of the SLNs. Also, Kalhapure et al. (2014), reported that vancomycin loaded SLN had a higher antibacterial effect against *S. aureus* than free vancomycin. SLNs also improved the antibacterial effect of florfenicol (Wang et al., 2015) and retinoic acid and lauric acid against *S. aureus* (Silva et al., 2015). The advantage of the SLNs are protection the drug from degradation, decrease the toxicity of the drug, have ability to payload the lipophilic and hydrophilic drug, easy to prepare, and have long-term stability; however they have disadvantages as inherent low drug loading capacity due to the crystalline structure of the solid lipid, the higher incidence of the polymorphic transition and unpredictable agglomeration (Mukherjee et al., 2009; Patidar et al., 2010; Kaur & Slavcev, 2013).

### 4.4. Nanogels

Among various nanoparticles, nanogels are a novel, innovative three-dimensional cross-linked nanocarrier with size ranges from 20 to 200 nm used in the drug delivery to release drug with a different mechanism such as PH-responsive, thermosensitive, enzyme-responsive and photoisomerization mechanisms at the target site (Table 2). Nanogels are taken into consideration over other drug carrier systems for some of the motives as.

They characterized by excessive biocompatibility, which helps them to be a completely promising approach for drug delivery systems (Sultana et al., 2013) and excessive biodegradability that is essential to prevent the accumulation of nanogel in the organs. Nanogels don’t have any immunological effect in the body due to they are inactive in the bloodstream and internal aqueous surroundings (Rigogliusoa et al., 2012). As well as, nanogels can be taken by different routes involving “oral, nasal, parenteral, pulmonary, intra-ocular and topical” methods of administration.

Prolonged serum half-life attributable to its tremendously smaller size enhances the invasion capability and prevents the rapid elimination by the kidney (Sultana et al., 2013). Furthermore, the nanogel is reflected as an ideal tool for transport the drug intracellular, and rapid responsiveness to ecological changes as (temperature and pH) resulted from “its ability to elude clearance by phagocytic cells and uptake with the assistance of reticuloendothelial organs, improved penetration into diseased sites as (solid tumors, inflamed tissue, and infarcted areas) in addition, its ability to enter the blood-brain barrier and carrying the drug safely into the cytoplasm of target cells”.

Nanogels are appropriate to be administered either hydrophilic or hydrophobic drugs. These affected by the kind of active groups that present in the polymer chains network, the crosslinking density, and the crosslinking agent involved in the polymeric system. Furthermore, nanogels are considered ideal applicants for the uptake and transport of (peptides, proteins, bio-macromolecules, and bulk drugs) due to they are accompanying with aqueous solutions, resulting in their capability to absorb water when located in an aqueous intermediate (Rigogliusoa et al., 2012).

The loading aptitude of the drug in nanogels is moderately high when compared to other nanocarriers and drug transport systems. The process of nanogels formulating is very effective for the reason that the drug is not wished in the first steps of the manufacturing process and can be presented to the nanogel in subsequent steps as soon as the nanogel distended with water and biological fluids. As well as, incorporating the agent into the nanogels is simple, impulsive, and does not fundamentally need any chemical responses (Soni & Yadav, 2016).

Nanogels are organized to be proficient for releasing the drug in a controlled and continuous form at the target place without any adverse reactions (Soni & Yadav, 2016). The action of bio-macromolecules can be positively enhanced and prolonged in the natural surroundings by incorporating in the nanogels. Nanogels can be expressed in the form of polymeric micellar nanogel systems that display slower patterns of dissociation, high stability over the surfactant micelles, and increasing the withholding period of loaded drugs (Sultana et al., 2013).

There are several types from nanogels used in mastitis diseases; for example, Krishna et al. (2017), confirmed that nano copper gel used as a therapy in clinical mastitis. Also, the red blood cells (RBC) nanogels were proved to neutralize MRSA-related toxins in the extracellular environment and stimulated bacterial phagocytosis by macrophages (Zhang et al., 2017) and also, PLGA nanoparticles loaded RBCs hydrogel neutralize *S. aureus* toxins (Wang et al., 2015); as well as, dextran cross-linked polyacrylamide nanogels loaded with zinc nitrate as an antibacterial agent against MRSA (Malzahn et al., 2014) were studied; as well as, silver nanoparticles loaded-dextran lysozyme nanogel showed higher antibacterial effect against *S. aureus* (Ferrer et al., 2014). The study reported by Mohammed et al. (2018) summarized that vancomycin loaded with carbapol nanogel by swelling deswelling mechanism, was released at an acidic PH of the inflamed tissue resulted from *S. aureus* infection, and its therapeutic efficacy was increased. Also, rosemary essential oils loaded-chitosan benzoic acid nanogel had an antimicrobial effect against *S. aureus* (Mohsenabadi et al., 2018) as the same result of gentamycin sulfate loaded chitosan nanogel (Wu et al., 2014) and vancomycin loaded mannose hydrogel that had anti-MRSA effect (Xiong et al., 2012). Silver nanoparticles loaded (poly-N-Iso-propyl acrylamide nanogel (Qasim et al., 2018), polyacrylic acid (Choi et al., 2013), Alginate nanocomposite hydrogel (Stojkovska et al., 2014), Acrylamide-methylpropane hydrogel (Boonkaew et al., 2014), and Fumaric acid cross linked-carboxy methyl acetate hydrogel (Bozaci et al., 2015) exhibited a potent bactericidal effect against *S. aureus* infection.

### 4.5. Inorganic metal nanoparticles

Metal nanoparticles can also be considered as antibacterial and antibiofilm. For example, silver nanoparticles (AgNPs) were used in subclinical mastitis (Dehkordi et al., 2011). They were deemed alternate to a highly expensive antimicrobial solution due to they have bactericidal and fungicidal effects through different actions as; damage cell membranes, protein denaturation, increasing of reactive oxygen species, interference with; DNA replication, proteins and enzymes expression (Li et al., 2014). Recently, AgNPs have shown antibacterial performance against *S. aureus* and also are incredibly effective against methicillin-resistant *S. aureus* (MRSA) (Wady et al., 2014). The combination of AgNPs and antibiotics were also estimated, erythromycin is as an example, combining with AgNPs against *S. aureus* (Kazemi et al., 2014). Moreover, selenium is a critical micronutrient, which has been inspected for several medical applications such as antibacterial, antioxidant, anti-inflammatory, and anti-cancer growth. Wang et al. (2018), indicated that selenium ameliorates inflamed udder epithelial cells caused by *S. aureus*, through preventing the action of nuclear factor kappa protein (NF-κB), mitogen-activated protein kinase (MAPK) and toll-like receptor 2 (TLR2) signaling pathways and inducing microRNA-146a (Sun et al., 2017). Also, nitric oxide nanoparticles were used in bovine mastitis treatment by combating *S. aureus* infection and overcoming the drawbacks of bacterial resistance (Cardozo et al., 2014).

Biofilms are an exact mechanism of MRSA persistence and antibacterial resistance for which nanoscale approaches can offer a novel tool to fight infections. Metal nanoparticles are a broad field of attention to prevent MRSA infections development (Hibbitts & O’Leary, 2018). The common metals nanoparticles which have a robust antimicrobial feature, representing an ability to eliminate MRSA biofilms (Mocan et al., 2014; Vijayakumar et al., 2015; Ferreira et al., 2016; Alhadrami & Al-Hazmi, 2017; Aswathanarayan & Vittal, 2017; Guo et al., 2017; Hsueh et al., 2017; Mekkawy et al., 2017), as explained in (Table 3).

**Table 3.** Metal nanoparticles for methicillin-resistant (MRSA) treatment.

Type	Mode of action	References
“Ag NPs”	Disturbance to the cell membrane of bacteria Inhibit transport of cytochrome and electron Binding with DNA/RNA and inhibiting their replication Binding with ribosome and inhibiting of protein synthesis Creation of reactive oxygen species Inhibit the formation of the cell wall of gram-positive bacteria	(Mekkawy et al., 2017)
“ZnO NPs”	Disruption to the bacterial cell membrane Formation of reactive oxygen species	(Vijayakumar et al., 2015; Aswathanarayan & Vittal, 2017)
“Cu/CuO NPs.”	Interacts with (amine and carboxyl) groups on the bacterial cell surface Formation of reactive oxygen species	(Hsueh et al., 2017)
“TiO_2_ NPs”	Photocatalysis process by UV stimulation leading to ROS formation	(Alhadrami & Al-Hazmi, 2017)
“MgX_2_/MgO NPs”	Inhibition to enzymes, ROS creation MgO-encouraged halogen adsorption	(Guo et al., 2017)
“Au NPs”	Their activity achieved through functionalization or combination therapy	(Mocan et al., 2014)
“Bi NPs”	“Radiation-stimulated formation of free radical and damage of DNA”	(Ferreira et al., 2016)

*Note*. Ag NPs: Silver Nanoparticles; ZnO: Zinc Oxide; Cu/CuO: Copper/Copper Oxide; TiO_2_: Titanium Oxide; MgX_2_: Magnesium with X_2_ referring to a bonded halide; Au: Gold; Bi: Bismuth.

Besides, Berni et al. (2013), tested violacein nanoparticles in mastitis disease, whereas violacein was considered a prevailing bactericidal agent, and its nanoparticles form were more efficient in contradiction of *S. aureus* than the ordinary agent. Yang et al. (2009), mentioned that amoxicillin nanoparticles were also active against *S. aureus*. As well as, Garg et al. (2015a), said that lasalocid showed substantial action against current mastitis-causing organisms besides MRSA and reported that the distribution rate of the nano-sized lasalocid exhibited faster than the microsized form in the udder. Therefore, the metallic nanoparticles have more advantages due to they easy to be prepared with different shapes and forms, having antibacterial actions and enhancing the drug stability, while they also have some disadvantages as releasing the metal ions in the medium leading to the cytotoxicity, accumulating in the body after their administration, and they may be agglomerated rapidly (Jijie et al., 2017)

## 5. Conclusions

*Staphylococcal* subclinical mastitis is a multifactorial and economically losses disease in dairy farming. The therapy difficulty involves the rapid emergence of multidrug resistance, the possible development of continuous, chronic, and repeated infections by biofilm formation and facultative intracellular parasitism. These make mastitis a continual challenge and a topic of consideration by many research groups. It is clear that administration of unsuitable and excessive antibiotics in dairy herds therapy, leading to several problems as examples, increasing the risk of antibiotic resistance, decreasing antibacterial activities, and process for checking and extending antimicrobial function was prolonged **(**Oliver & Murinda, 2012**).** So, there is an urgent necessity to combat the limitations of traditional antibiotics.

Recently, advancements in nanoparticles with unique physiochemical properties and functionalization have produced a remarkable impact on overcoming the restrictions posed by antibiotics (Yah & Simate, 2015). Last few years, several different nanoparticles have been well-known for *Staphylococcal* infection therapy, we have briefly summarized the recent studies in this area in Tables 2 and 3. These nanoparticles display higher intracellular uptake than the other traditional form of drug delivery systems, increasing the accumulation and the retention time of the drug intracellular, improving the antibacterial activity of the drug, decreasing the antimicrobial resistance, and inhibiting the biofilm formation. Therefore, shifting our opinion toward the nanoworld can overcome and challenge the treatment difficulties which accompanied by the *S.aureus* mastitis.

## 6. Future perspectives for mastitis treatment

Facing the therapeutic challenges of the *S. aureus* mastitis disease, we still need to discover and fabricate new safe, costly, and effective nanoformulations against the *S. aureus* mastitis. As we mentioned before, *S. aureus* can invade the tissue and reside intracellularly in special compartments. So, the efficacy of the nanoparticles must be strengthened to achieve better release the drug to the infected site and colocalization between the drugs and the intracellular *S. aureus* (Xie et al., 2014). Recently, our research team prepared self-assembly tilmicosin nanogel by a combination of (SLN) technology with in-situ hydrogel technology to improve the treatment effect of tilmicosin against *S. aureus* cow mastitis (Zhou et al., 2019). Moreover, nowadays we are trying to improve our work by designing different preparations from the nanogel to overcome cow mastitis. Also, further investigations on the lines of stimuli-responsive nanogel are necessary to be prepared within the improvement of a topical nanogel counter to medical mastitis. Although the great potential of drug delivery by nanoparticles, there are some obstacles facing them like the immature release of the loaded drug before the specific lesion, rapid clearance of drugs from the body, and phagocytosis by the immune cells. These problems can be solved via a combination of nanomaterials with the natural drug delivery system by coating nanomaterials with cell membranes of natural cells such as stem cells, Red blood cells, platelets, and bacterial cells considered smart drug delivery systems. They allow accumulation of the drug for a long time in the circulation and penetration through the cell membrane to prevent intracellular infection. In addition to these merits, RBCs have the ability to relieve the destruction caused by the bacterial infection via neutralization of the bacterial toxin; as well as, stem cells would offer a promising attitude for tissue repair and increase therapeutic efficacy in cows mastitis in the future.

To translate the nanomaterials from the laboratory to the clinics, they need a lot of efforts, time and guidelines for their large scale production, our groups recently have provided wide effective techniques for SLNs and nanocrystal nanosuspension, which will be beneficial for their application.

Finally, there has been plenty of enthusiasm to progress nanorobots that may be used in tissue diagnosis and restoration mechanism. These have not nevertheless used before and remain futuristic studies that possibly could be succeeded by human-made within the very near future (Zhou et al., 2018).
